# Association between smoking behavior and oral health problems: A national cross-sectional study in Korea

**DOI:** 10.18332/tid/200693

**Published:** 2025-03-07

**Authors:** Ju Yeon Lee, Chae Heon Song, Jaewoo Kim, Yun Seo Jang, Eun-Cheol Park

**Affiliations:** 1 Medical Courses, Yonsei University College of Medicine, Seoul, Republic of Korea; 2 Pre-Medical Courses, Yonsei University College of Medicine, Seoul, Republic of Korea; 3 Institute of Health Services Research, Yonsei University, Seoul, Republic of Korea; 4 Graduate School of Public Health, Yonsei University, Seoul, Republic of Korea; 5 Department of Preventive Medicine and Public Health, College of Medicine, Yonsei University, Seoul, Republic of Korea

**Keywords:** smoking, oral health, smoking cessation, electric cigarette, smoking behavior

## Abstract

**INTRODUCTION:**

Smoking is a risk factor that significantly affects general and oral health by altering the oral environment, increasing plague build-up, and reducing blood flow in the gums, leading to tooth decay and periodontal disease. Therefore, this study investigated the association between smoking behaviors, such as smoking duration and cessation, and oral health problems.

**METHODS:**

This study analyzed a secondary dataset of the Korea National Health and Nutrition Examination Survey (2019–2021) that included 6150 men and 7574 women. Individuals were classified as current smokers if they were currently smoking regular cigarettes, heated tobacco products, or electronic cigarettes. Oral health problems included toothaches and chewing difficulties. Multiple logistic regression was used to calculate adjusted odds ratios (AORs) and 95% confidence intervals (CIs) for the association between smoking behavior and oral health problems.

**RESULTS:**

Among participants of both sexes, current smokers had more oral health problems compared with non-smokers (men, AOR=1.60; 95% CI: 1.35–1.89; women, AOR=1.91; 95% CI: 1.33–2.71), as did ex-smokers (men, AOR=1.39; 95% CI: 1.18–1.63; women, AOR=1.47; 95% CI: 1.18–1.83). The longer the smoking cessation period, the lower was the prevalence of oral health problems. Additionally, high pack-years were associated with oral health issues. Regular cigarettes were more likely to cause problems than e-cigarettes (men, AOR=1.56; 95% CI: 1.31–1.86; women, AOR=1.96; 95% CI: 1.53–2.52), while vaping (men, AOR=1.36; 95% CI: 1.05–1.74; women, AOR=1.64; 95% CI: 1.06–2.53) and dual smoking (men, AOR=1.57; 95% CI: 1.14–2.16; women, AOR=1.97; 95% CI: 1.10–3.50) were also associated with oral health issues.

**CONCLUSIONS:**

This study confirmed that smoking is strongly associated with oral health problems. Public health efforts should focus on prevention and tailored interventions to support quitting and improve oral health outcomes in both current and ex-smokers.

## INTRODUCTION

Smoking significantly impacts human health, accounting for 24% and 7% of all deaths among men and women, respectively, in developed countries, and leading to an average life expectancy reduction of approximately 8 years^1^. In addition to reducing life expectancy, smoking has numerous other negative health effects, including systemic inflammatory responses^2^; osteoporotic fractures^3^; increased risks of lung, oral, laryngeal, bladder, renal, pancreatic, gastric, cervical, hepatic, penile, and colorectal cancers; and increased risks of coronary artery, cerebrovascular, and peripheral vascular diseases^4^. Additionally, smoking has a significant impact on oral health, with smokers experiencing dental pain, orofacial pain^5^, tooth loss, and periodontitis^6,7^. Given that permanent teeth do not regenerate once they have grown^8^, maintaining good oral health is of utmost importance^9^.

Prolonged exposure to smoking leads to an increase in the acidity of the saliva, reducing its buffering capacity and thereby hindering the natural cleansing process in the mouth. Consequently, excessive plaque build-up occurs, which increases the incidence of dental caries^10^. Furthermore, smoking stimulates the release of epinephrine and constricts peripheral blood vessels, reducing blood flow to the gums, and contributing to the development of periodontal disease^11^. Additionally, smoking impairs neutrophil function and reduces the serum antibody response against bacteria that cause periodontal disease, significantly affecting oral health^12^. Repeated exposure to electronic cigarettes can cause inflammation of oral epithelial cells and accelerate aging, thereby increasing the risk of oral diseases^13^. Therefore, evaluating the impact of e-cigarettes and dual smoking on oral health is essential. Although many existing studies have focused on regular cigarette smokers, research on the association between oral health and vaping, pack-years, and duration of smoking cessation in ex-smokers is lacking. Additionally, oral health symptoms, such as tooth loss, toothache, periodontitis, and chewing difficulties, have been examined^14-17^. However, as patients experiencing one oral health issue often tend to have other related problems, conducting a detailed analysis to account for overlapping cases of oral health issues is necessary.

Therefore, this study aimed to examine the association between smoking behavior and oral health problems in adults. Our findings offer a comprehensive understanding of the association between various smoking behaviors, such as smoking frequency/amount and cessation duration, and their specific associations with oral health. Additionally, this study aimed to contribute to the development of targeted interventions and strategies to improve oral health in both current and ex-smokers.

## METHODS

### Data and study population

This study analyzed a secondary dataset from the Korea National Health and Nutrition Examination Survey (KNHANES), spanning 3 years from 2019 to 2021, conducted by the Korea Centers for Disease Control and Prevention Agency^18^. The KNHANES is a statutory survey on health behaviors, prevalence of chronic diseases, and dietary and nutritional intake status of the population, conducted based on Article 16 of the National Health Promotion Act^18^. The purpose of the survey was to generate nationally representative and reliable statistics on the health status, health behaviors, and dietary and nutritional intake of the population, and to use these data as a basis for setting and evaluating the goals of the National Health Promotion Comprehensive Plan, developing health promotion programs, and informing health policies.

The KNHANES collects data through household member verification, health interview, health examination, and nutrition surveys. The sampling frame for the KNHANES is based on the most recent Population and Housing Census data available at the time of sample design, which allows for the extraction of a representative sample of the target population comprising all residents aged ≥1 year in the Republic of Korea. The household member verification survey is a preliminary survey conducted to identify all dwelling units and households within the selected sample areas, and to select households (and their members) to participate in the health interview, examination, and nutrition surveys. This survey updates information on the target areas and households since the construction of the sampling frame, enabling the selection of current survey participants based on the updated information. Household member verification survey data are also used to schedule visits to mobile examination centers (for the nutrition survey, household visits are conducted), distribute survey results, and calculate response rates and sampling weights^18,19^. The respondents completed the questionnaires, and all collected data were anonymized. Since the KNHANES adheres to the Declaration of Helsinki and offers publicly accessible data, ethical approval was not necessary.

**Figure 1:**
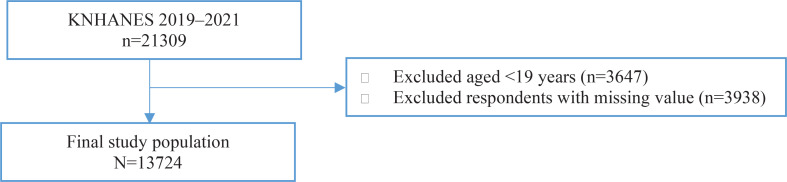
Flowchart displaying the inclusion and exclusion of study participants from 2019 to 2021

Of the 21309 survey participants, those aged <19 years and those who did not participate in the KNHANES smoking questionnaire were excluded (n=3647). Participants with missing data were also excluded (n=3938). Consequently, a final sample of 13724 participants, including 6150 men and 7574 women, were analyzed (Figure 1).

### Variables

The dependent variable was oral health problems, evaluated through self-reported questions. We considered individuals to have oral health problems if they had any one of toothache and chewing difficulties. Toothache was defined as pain, throbbing, or aching in the teeth over the past year, or pain in the teeth when consuming hot or cold beverages or foods. Chewing difficulties were defined as experiencing discomfort while chewing food due to issues with the teeth, dentures, gums, or other oral problems.

Individuals were classified as current smokers if they were currently smoking any of the following^20-23^: regular cigarettes, heated tobacco products, or electronic cigarettes. Ex-smokers were those who had smoked in the past, but did not smoke any of these products. Non-smokers were defined as those who had never smoked any of the aforementioned products. Also, current smokers were defined as regular cigarette smokers if they smoked only regular cigarettes; e-cigarette smokers, only vaping; and dual smokers, both types of cigarettes.

All analyses were conducted while controlling covariates, including demographic (age, region), socioeconomic (marital status, education level, household income, occupational classification), health-related (current alcohol consumption status, physical activity level, body mass index, diagnosis of hypertension or diabetes), and other factors (survey year). Covariates were selected based on biological plausibility, previous literature, and their potential to confound the association between smoking behavior and oral health problems.

### Statistical analysis

Descriptive analysis was employed to examine the general characteristics of the sample population and reported as frequencies (n) and percentages (%). The chi-squared test was used to evaluate and compare the general characteristics of the study population. Subsequently, a multiple logistic regression analysis was performed to investigate the association between smoking behavior and oral health problems.

Subgroup analysis stratified occupation categories to demonstrate the association between the occupation of current smokers or ex-smokers and their oral health problems. Additionally, a subgroup analysis stratified by smoking type, cessation status, and pack-years was employed to elucidate the association between oral health problems and the type of cigarettes smoked, smoking cessation duration for ex-smokers, and smoking duration for current smokers. Finally, we performed an additional analysis by categorizing and examining each symptom of oral health problems in detail. This allowed us to conduct a more granular assessment of individual issues. Furthermore, a significant interaction effect was observed between sex and smoking status (p=0.0294), indicating that the association between smoking behavior and oral health problems differed by sex. Given this significant interaction, we conducted sex-stratified analyses to further explore these differences. In this study, all estimates were calculated using sample weighting procedures, with clusters and strata assigned to the participants. The results are presented as adjusted odds ratios (AORs) with 95% confidence intervals (CIs). Statistical significance was assessed using two-sided p-values, with a threshold of p<0.05. All analyses were conducted using SAS version 9.4 (SAS Institute Inc., Cary, NC, USA), with statistical significance set at a p<0.05.

## RESULTS

The characteristics of the study population are presented in Table 1. Of the 13723 participants, 6150 were men and 7573 were women; among them, 2211 (36.0%) men and 2903 (38.3%) women reported having oral health problems. Among the men with oral health problems, 424 were non-smokers (19.1%), 974 were ex-smokers (44.1%), and 813 were current smokers (36.8%); among the women with oral health problems, 2472 were non-smokers (85.1%), 210 were ex-smokers (7.2%), and 224 were current smokers (7.7%). The chi-squared test revealed a significant association between smoking status and oral health problems in participants of both sexes (p<0.0001).

**Table 1:** General characteristics of the study population in a secondary analysis of the KNHANES dataset, 2019–2021 (N=13723)

*Variables*	*Oral health problems*													
	*Male*							*Female*						
	*Total*		*Yes*		*No*		*p*	*Total*		*Yes*		*No*		*p*
	*n*	*%*	*n*	*%*	*n*	*%*		*n*	*%*	*n*	*%*	*n*	*%*	
	*6150*	*100*	*2211*	*36.0*	*3939*	*64.0*		*7573*	*100*	*2903*	*38.3*	*4670*	*61.7*	
**Smoking behavior**							<0.0001							<0.0001
Non-smoker	1539	25.0	424	27.6	1115	72.4		6638	87.7	2472	37.2	4166	62.8	
Ex-smoker	2500	40.7	974	39.0	1526	61.0		490	6.5	210	42.9	280	57.1	
Current smoker	1771	28.8	707	39.9	1064	60.1		445	5.9	221	49.7	224	50.3	
**Age** (years)														
20–29	831	13.5	182	21.9	649	78.1		801	10.6	230	28.7	571	71.3	
30–39	815	13.3	220	27.0	595	73.0		961	12.7	288	30.0	673	70.0	
40–49	1077	17.5	324	30.1	753	69.9		1344	17.7	428	31.8	916	68.2	
50–59	1063	17.3	448	42.1	615	57.9		1480	19.5	564	38.1	916	61.9	
60–69	1150	18.7	510	44.3	640	55.7		1475	19.5	638	43.3	837	56.7	
≥70	1214	19.7	527	43.4	687	56.6		1512	20.0	755	49.9	757	50.1	
**Marital status**							<0.0001							0.0019
Married	4275	69.5	1606	37.6	2669	62.4		4886	64.5	1810	37.0	3076	63.0	
Divorced/separated	289	4.7	146	50.5	143	49.5		504	6.7	234	46.4	270	53.6	
Single/widow	1586	25.8	459	28.9	1127	71.1		2183	28.8	859	39.3	1324	60.7	
**Education level**							<0.0001							<0.0001
Middle school or lower	1354	22.0	651	48.1	703	51.9		2477	32.7	1236	49.9	1241	50.1	
High school	2271	36.9	796	35.1	1475	64.9		2401	31.7	826	34.4	1575	65.6	
College or higher	2525	41.1	764	30.3	1761	69.7		2695	35.6	841	31.2	1854	68.8	
**Household income**							<0.0001							<0.0001
Low	1023	16.6	468	45.7	555	54.3		1519	20.1	758	49.9	761	50.1	
Mid-low	1476	24.0	581	39.4	895	60.6		1854	24.5	734	39.6	1120	60.4	
Mid-high	1697	27.6	581	34.2	1116	65.8		2024	26.7	714	35.3	1310	64.7	
High	1954	31.8	581	29.7	1373	70.3		2176	28.7	697	32.0	1479	68.0	
**Region**							<0.0001							<0.0001
Metropolitan	2568	41.8	822	32.0	1746	68.0		3345	44.2	1161	34.7	2184	65.3	
Urban	2229	36.2	800	35.9	1429	64.1		2675	35.3	1068	39.9	1607	60.1	
Rural	1353	22.0	589	43.5	764	56.5		7573	100.0	674	8.9	879	11.6	
**Occupation category**							<0.0001							<0.0001
White-collar	1683	27.4	490	29.1	1193	70.9		1652	21.8	514	31.1	1138	68.9	
Pink-collar	649	10.6	196	30.2	453	69.8		1137	15.0	421	37.0	716	63.0	
Blue-collar	2022	32.9	841	41.6	1181	58.4		1181	15.6	520	44.0	661	56.0	
Unemployed	1796	29.2	684	38.1	1112	61.9		3603	47.6	1448	40.2	2155	59.8	
**Current alcohol consumption status**							<0.0001							<0.0001
Never or occasionally	1984	32.3	760	38.3	1224	61.7		4607	60.8	1871	40.6	2736	59.4	
2–4 times/month	2184	35.5	683	31.3	1501	68.7		2164	28.6	744	34.4	1420	65.6	
2–4 times/week	1982	32.2	768	38.7	1214	61.3		802	10.6	288	35.9	514	64.1	
**Physical activity**							<0.0001							<0.0001
Adequate	2832	46.0	943	33.3	1889	66.7		2970	39.2	1038	34.9	2932	98.7	
Inadequate	3318	54.0	1268	38.2	2050	61.8		4603	60.8	1865	40.5	2738	59.5	
**BMI**							0.055							<0.0001
Underweight	158	2.6	70	44.3	88	55.7		413	5.5	152	36.8	261	63.2	
Normal	1724	28.0	634	36.8	1090	63.2		3298	43.5	1177	35.7	2121	64.3	
Overweight	1598	26.0	589	36.9	1009	63.1		1550	20.5	590	38.1	960	61.9	
Obesity of stage 1	2251	36.6	767	34.1	1484	65.9		1866	24.6	789	42.3	1077	57.7	
Obesity of stage 2 and 3	419	6.8	151	36.0	268	64.0		446	5.9	195	43.7	251	56.3	
**Diagnosis of hypertension**							<0.0001							<0.0001
Yes	1660	27.0	679	40.9	981	59.1		1852	24.5	857	46.3	995	53.7	
No	4490	73.0	1532	34.1	2958	65.9		5721	75.5	2046	35.8	3675	64.2	
**Diagnosis of diabetes**							<0.0001							<0.0001
Yes	722	11.7	321	44.5	401	55.5		745	9.8	365	49.0	380	51.0	
No	5428	88.3	1890	34.8	3538	65.2		6828	90.2	2538	37.2	4290	62.8	
**Survey year**							<0.0001							<0.0001
2019	2318	37.7	802	34.6	1516	65.4		2882	38.1	1047	36.3	1835	63.7	
2020	2215	36.0	760	34.3	1455	65.7		2673	35.3	982	24.0	1691	63.3	
2020	1617	26.3	649	40.1	968	59.9		2018	26.6	874	24.0	1144	56.7	

BMI: body mass index.

Table 2 presents the results of the multiple logistic regression analysis adjusted for all covariates and stratified by sex to examine the association between smoking status and oral health problems. Among men, ex-smokers and current smokers had AORs of 1.39 (95% CI: 1.18–1.63) and 1.60 (95% CI: 1.35–1.89), respectively, compared with non-smokers. Among women, ex-smokers and current smokers had AORs of 1.47 (95% CI: 1.18–1.83) and 1.91 (95% CI: 1.33–2.71), respectively, compared with non-smokers.

**Table 2:** Multiple logistic regression results showing the association between smoking behaviors and oral health problems, adjusted for covariates, based on the KNHANES dataset (2019–2021) (N=13723)

*Variables*	*Oral health problems*			
	*Male*		*Female*	
	*AOR*	*95% CI*	*AOR*	*95% CI*
**Smoking behavior**				
Non-smoker ®	1		1	
Ex-smoker	1.39	1.18–1.63	1.47	1.18–1.83
Current smoker	1.60	1.35–1.89	1.91	1.50–2.43
**Age** (years)				
20–29 ®	1		1	
30–39	1.41	1.04–1.91	1.10	0.85–1.41
40–49	1.45	1.07–1.95	1.26	0.97–1.64
50–59	2.23	1.64–3.03	1.51	1.17–1.96
60–69	2.24	1.61–3.11	1.56	1.18–2.07
≥70	1.80	1.25–2.58	1.69	1.25–2.29
**Marital status**				
Married ®	1		1	
Divorced/separated	1.19	0.87–1.62	1.04	0.83–1.31
Single/widow	0.99	0.79–1.23	0.97	0.83–1.14
**Education level**				
Middle school or lower ®	1		1	
High school	0.79	0.65–0.96	0.71	0.60–0.85
College or higher	0.75	0.61–0.93	0.71	0.58–0.86
**Household income**				
Low	1.72	1.36–2.18	1.28	1.03–1.58
Mid-low	1.37	1.13–1.66	1.09	0.93–1.27
Mid-high	1.46	1.04–1.23	1.08	0.93–1.26
High ®	1		1	
**Region**				
Metropolitan ®	1		1	
Urban	1.26	1.05–1.52	1.23	1.05–1.46
Rural	1.50	1.23–1.84	1.15	0.94–1.41
**Occupation category**				
White-collar	0.99	0.80–1.23	1.01	0.85–1.18
Pink-collar	0.88	0.69–1.14	0.98	0.83–1.16
Blue-collar	1.41	1.01–1.19	1.04	0.88–1.22
Unemployed ®	1		1	
**Current alcohol consumption status**				
Never or occasionally ®	1		1	
2–4 times/month	0.86	0.72–1.02	0.89	0.78–1.02
2–4 times/week	1.01	0.86–1.19	0.94	0.77–1.15
**Physical activity**				
Adequate ®	1		1	
Inadequate	0.93	0.81–1.06	1.06	0.94–1.19
**BMI**				
Underweight	1.63	1.02–2.61	1.27	0.97–1.67
Normal	1		1	
Overweight	1.12	0.94–1.33	0.94	0.80–1.10
Obesity of stage 1	0.91	0.78–1.07	1.07	0.93–1.24
Obesity of stage 2 and 3	1.09	0.82–1.45	1.21	0.95–1.54
**Diagnosis of hypertension**				
No ®	1		1	
Yes	1.03	0.88–1.20	1.12	0.92–1.36
**Diagnosis of diabetes**				
No ®	1		1	
Yes	1.17	0.96–1.42	1.07	0.89–1.27
**Survey year**				
2019 ®	1		1	
2020	1.06	0.88–1.27	1.07	0.89–1.27
2021	1.42	1.16–1.74	1.40	1.17–1.67

AOR: adjusted odds ratio. Adjusted for all covariates: age, marital status, education level, household income, region, occupation category, current alcohol consumption status, physical activity, body mass index (BMI), diagnosis of hypertension, diagnosis of diabetes and survey year. ® Reference categories.

Table 3 presents the subgroup analysis stratified by occupation categories. Among both men and women in the pink-collar group, a strong association was observed between both ex-smoking and current smoking and oral health problems (ex-smokers: AOR=1.65; 95% CI: 0.87–3.11; current smokers: AOR=1.70; 95% CI: 0.99–2.93). Among current smokers, those unemployed showed a significant association with oral health problems. Additionally, among women, current smokers in the white-collar group demonstrated higher AORs.

**Table 3:** Subgroup analysis results stratified by occupational categories and adjusted for all covariates, using data from the KNHANES dataset (2019–2021) (N=13723)

*Occupation category*	*Oral health problems*									
	*Male*					*Female*				
	*Nonsmoker ®*	*Ex-smoker*		*Current smoker*		*Nonsmoker ®*	*Ex-smoker*		*Current smoker*	
	*AOR*	*AOR*	*95% CI*	*AOR*	*95% CI*	*AOR*	*OR*	*95% CI*	*AOR*	*95% CI*
White-collar	1	1.36	0.99–1.87	1.59	1.16–2.17	1	1.42	0.96–2.11	1.96	1.21–3.20
Pink-collar	1	1.65	0.87–3.11	1.70	0.99–2.93	1	2.56	1.41–4.65	2.53	1.40–4.58
Blue-collar	1	1.33	0.99–1.80	1.56	1.15–2.10	1	2.22	0.95–5.18	1.57	0.78–3.15
Unemployed	1	1.32	0.96–1.81	1.67	1.17–2.39	1	1.15	0.84–1.59	1.80	1.25–2.58

AOR: adjusted odds ratio. Adjusted for all covariates: age, marital status, education level, household income, region, occupation category, current alcohol consumption status, physical activity, body mass index (BMI), diagnosis of hypertension, diagnosis of diabetes and survey year. ® Reference category.

Table 4 presents the results of the subgroup analysis stratified by smoking type, cessation status, and pack-years. When analyzing the association based on smoking type, among both men and women, the odds of oral health problems were high for regular cigarette smokers and e-cigarette smokers; however, regular cigarette smokers had higher AORs than e-cigarette smokers (regular cigarette smokers: men, AOR=1.56; 95% CI: 1.31–1.86; women, AOR=1.96; 95% CI: 1.53–2.52; e-cigarette smokers: men, AOR=1.36; 95% CI: 1.05–1.74; women, AOR=1.64; 95% CI: 1.06–2.53). Among ex-smokers, participants of both sexes showed decreased ORs for oral health problems as the duration of smoking cessation increased (<10 years: men, AOR=1.55; 95% CI: 1.26–1.90; women, AOR=1.43; 95% CI: 1.03–1.98; ≥30 years: men, AOR=1.19; 95% CI: 0.89–1.58; women, AOR=1.05; 95% CI: 0.54–2.02). Furthermore, an analysis of pack-years among ex-smokers and current smokers (excluding non-smokers) demonstrated a clear correlation, with oral health problems increasing as pack-years accumulated (<10 years: men, AOR=1.31; 95% CI: 1.09–1.57; women, AOR=1.66; 95% CI: 1.37–2.00; ≥20 years: men, AOR=1.88; 95% CI: 1.56–2.26; women, AOR=2.10; 95% CI: 1.19–3.73).

**Table 4:** Subgroup analysis results examining smoking type, cessation status, and pack-years, adjusted for covariates, based on the KNHANES dataset (2019–2021) (N=13723)

*Variables*	*Oral health problems*			
	*Male*		*Female*	
	*AOR*	*95% CI*	*AOR*	*95% CI*
**Smoking type** (considering e-cigarettes)				
Non-smoker ®	1		1	
Regular cigarette smoker	1.56	1.31–1.86	1.96	1.53–2.52
E-cigarette smoker	1.36	1.05–1.74	1.64	1.06–2.53
Dual smoker	1.57	1.14–2.16	1.97	1.10–3.50
**Smoking cessation** (years)				
Non-smoker ®	1		1	
Ex-smoker (≥30)	1.19	0.89–1.58	1.05	0.54–2.02
Ex-smoker (≥20 and <30)	1.23	0.96–1.58	1.46	0.86–2.47
Ex-smoker (≥10 and <20)	1.34	1.07–1.68	1.76	1.18–2.63
Ex-smoker (<10)	1.55	1.26–1.90	1.43	1.03–1.98
Current smoker	1.61	1.36–1.90	1.92	1.51–2.44
**Pack-years**				
Non-smoker ®	1		1	
<10	1.31	1.09–1.57	1.66	1.37–2.00
≥10 and <20	1.44	1.18–1.76	1.63	1.12–2.38
≥20	1.88	1.56–2.26	2.10	1.19–3.73

AOR: adjusted odds ratio. Adjusted for all covariates: age, marital status, education level, household income, region, occupation category, current alcohol consumption status, physical activity, body mass index (BMI), diagnosis of hypertension, diagnosis of diabetes and survey year. ® Reference category.

**Figure 2:**
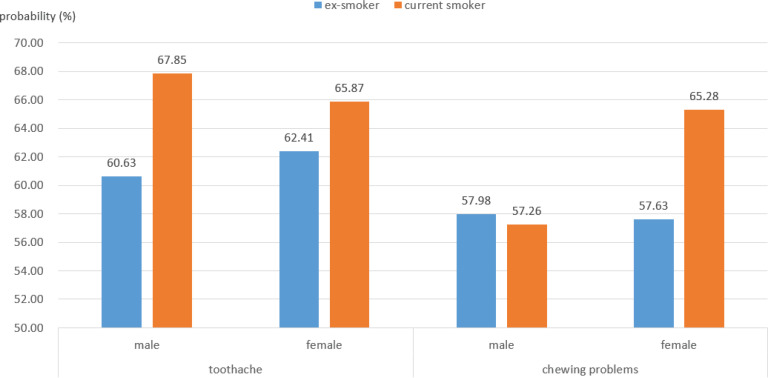
Predicted probabilities of oral health problems by smoking status, stratified by type of oral health conditions

Figure 2 presents the subgroup analysis of the causes of oral health issues using predicted probabilities. Among both men and women, current smokers exhibited a higher probability of experiencing toothache compared to ex-smokers. The estimated probability of toothache among male current smokers was 67.85%, compared to 60.63% for male ex-smokers. Similarly, female current smokers had a 65.87% probability of experiencing toothache, whereas female ex-smokers had a probability of 62.41%. Regarding chewing problems, the probabilities varied by smoking status and sex. Among men, ex-smokers had a slightly higher probability of chewing problems (57.98%) compared to current smokers (57.26%). However, among women, current smokers had a significantly higher probability of experiencing chewing problems (65.28%) compared to ex-smokers (57.63%). These results indicate a significant association between smoking and oral health issues, particularly for current smokers, with greater differences observed in women for chewing problems.

## DISCUSSION

This study found that smoking increased the risk of oral health problems in individuals of both sexes. These results support previous studies indicating that cigarette smoking and other types of tobacco use can cause various oral health problems, including bleeding upon brushing, tooth stains, bad breath, and oral cancer^24,25^. According to previous findings, individuals with a history of smoking have a higher incidence of oral health problems than non-smokers, and current smokers are at an even greater risk than ex-smokers. This is in line with previous studies showing that the cessation of tobacco use has a beneficial effect on the outcomes of periodontal therapy and halts the progression of periodontal disease^26^. Therefore, smoking cessation is necessary to prevent oral health problems.

In addition, the risk of oral health problems was found to be higher in female than male participants. Both current and ex-smokers among female participants exhibited a greater susceptibility to oral health problems. For instance, studies have shown that women have more difficulty maintain long-term smoking cessation, as stress and adverse life events affect their ability to quit^27^. Research also suggests that women are more likely than men to continue smoking in the presence of financial hardship and are less likely to quit smoking in response to health-related issues^28^. As women find it more difficult to quit smoking, compared with men, special attention, including stress management, is required. However, this finding does not imply that male smokers disregard oral health issues. Evidence from previous studies indicates that men are more likely to ignore their oral health and often delay visiting dentists until faced with acute problems^29^. This behavior contributes to a higher male-to-female ratio for oral cancer, largely due to more tobacco use among men^29^. Thus, initiatives aimed at improving oral health awareness and preventive care among male smokers are crucial.

According to the results of the independent subgroup analysis, individuals in pink-collar jobs within the service industry demonstrated a relatively stronger association between smoking and oral health problems than those in other occupations. This is consistent for both sexes, as well as for both current and ex-smokers. From the present study’s findings, this is hypothesized to be due to an increased likelihood of smoking due to job-related stress^24^, as pink-collar workers, such as teachers, are under a high level of stress^30^. These results further suggest that among current smokers, unemployment correlates with oral health problems. Additionally, among female current smokers, those in the white-collar group had higher ORs.

Significant results were also observed in the analysis of associations based on smoking type, duration of smoking cessation, and pack-years. The group that smoked both e-cigarettes and regular cigarettes had a higher risk of oral health problems than did smokers who only smoked one of the two types of cigarettes. Furthermore, e-cigarettes have a negative effect on oral health^31-33^; however, vapor use may be considered a healthier alternative to cigarette smoking in terms of periodontal health^34^. Additionally, pain in the teeth and gums is more often perceived by cigarette smokers than by electronic cigarette smokers and non-smokers^35^. Considering this, people who smoke only vapor are healthier than those who smoke regularly. For dual smokers, quitting both cigarettes, or only regular cigarettes, is likely to be effective for oral health. The longer the ex-smokers abstained from smoking, the better was their oral health; moreover, smokers had poorer oral health and longer pack-year history. This finding supports the idea that early smoking cessation reduces the risk of oral health problems.

Finally, the analysis conducted by stratifying the relationship between smoking and oral health problems by disease type revealed significant results. Patients with toothache were more strongly affected by smoking than those without toothache. This is in line with a previous study demonstrating that current smokers were at an increased risk of experiencing toothache in the past 6 months^36^. A similar trend was observed for chewing problems. Among women, current smoking showed a stronger association with chewing problems. In addition to the oral health problems addressed in this study, many other diseases are strongly associated with smoking^24,25^.

### Strengths and limitations

This study has several strengths. First, we utilized data from the KNHANES, a nationally representative survey reflecting the characteristics of South Koreans and their health behaviors. The survey used random cluster sampling, to allow statistical generalization of the research findings to all the population. Second, the inclusion of recent data (2019–2021) is significant, encompassing current smoking status, e-cigarette use, smoking history, and cessation attempts and plans. Third, while previous studies examined the association between smoking status and oral health, focusing on regular cigarette smokers, the present study included e-cigarette smokers also. This study has some limitations. First, this study was cross-sectional, so a temporal relationship cannot be ascertained. Also, smoking may not have preceded oral health problems, so further prospective cohort studies are needed to confirm our findings. Second, the KNHANES uses self-report surveys, which can introduce issues of reliability and accuracy of health-related, socioeconomic, and smoking status information and recall bias that can potentially lead to the underestimation of smoking prevalence. Third, potentially residual confounding variables may remain, despite attempts to include as many independent variables related to smoking and oral health problems. Fourth, the overall state of oral health is not solely based on the presence of toothache or chewing problems; individuals may still have poor oral health due to other oral issues. Fifth, we only analyzed the presence and types of problems; owing to limitations in the KNHANES data, information on the degree or frequency of pain was not collected. Finally, the study findings are limited to South Korea, as data were exclusively from the KNHANES and differences may exist due to variations in cultural, socioeconomic, and healthcare factors. Thus, there is a need to include a more diverse, international population to enhance the validity of our findings to other countries.

## CONCLUSIONS

This study found that smoking negatively affects oral health. A comparison between ex-smokers and current smokers indicated that smoking cessation could reduce the risk of oral health problems. Therefore, the government should consider active smoking prevention efforts and try and implement smoking prevention activities for adults in educational settings. Dual smokers are recommended to quit both e-cigarettes and regular cigarettes. By implementing these factors, the oral health of smokers can be improved, and oral health problems can be mitigated.

## Data Availability

The data supporting this research are available from the Korea National Health and Nutrition Examination Survey website (https://knhanes.kdca.go.kr/knhanes/main.do).
